# A&E Waits for Mental Health Inpatient Beds

**DOI:** 10.1192/bjo.2026.11605

**Published:** 2026-06-30

**Authors:** Anna Poller, Bruce Tamilson

**Affiliations:** 1 City Saint George's University, London, United Kingdom; 2 South West London and St George's NHS Mental Health Trust, London, United Kingdom

## Abstract

**Aims:**

South West London and St George’s Mental Health (SWLStG) hospitals operate a 12-hour pathway for mental health patients (MHPs) requiring admission from the A&E. However, prolonged waits beyond this timeframe have been frequently observed, with some patients remaining in A&E for more than 72 hours following the decision to admit (DTA). This audit aimed to determine the proportion of MHPs waiting longer than 72 hours for a psychiatric inpatient bed from a district general hospital A&E over a 13-month period. Secondary aims were to establish the average waiting time for this cohort and to explore whether bed wait duration differed between detained and informal admissions.

**Methods:**

Hospital administrative records were retrospectively reviewed for all MHPs awaiting inpatient psychiatric admission from a district general hospital A&E between October 2024 and November 2025. Data collected included time of DTA and time of transfer to an inpatient psychiatric bed. Length of stay (LoS) was calculated from DTA to admission.

**Results:**

During the audit period, 642 patients were assessed as requiring psychiatric admission. Of these, 105 patients (16%) waited longer than 72 hours for an inpatient bed. The mean LoS for this cohort was 5 days, 23 hours, and 12 minutes, with a maximum LoS of 24days, 20 hours, and 39 minutes. Patients detained under the MHA (n=47) experienced longer waiting times compared with informal admissions (n=62), with an average additional delay of 4 hours and 38 minutes (mean LOS 6 days, 1 hour, 8 minutes vs 5 days, 20 hours, 30 minutes respectively).

**Conclusion:**

This audit demonstrates persistent difficulty in achieving timely psychiatric admission from A&E, with 16% of MHPs waiting over 72 hours following DTA. Patients with higher clinical acuity, reflected by detention under the MHA, experienced longer bed waits. Contributing factors are likely multifactorial, including reduced inpatient bed capacity, increasing mental health demand, limited community alternatives, and A&E environments that are poorly resourced for prolonged psychiatric care. Nationally, psychiatric inpatient bed numbers have reduced by approximately 73% over the past three decades, alongside a substantial rise in mental health presentations post Covid pandemic. Future work should replicate this audit across multiple A&E sites to better characterise systemic delays and inform service-level planning.

